# The Scales Project, a cross-national dataset on the interpretation of thermal perception scales

**DOI:** 10.1038/s41597-019-0272-6

**Published:** 2019-11-26

**Authors:** Marcel Schweiker, Amar Abdul-Zahra, Maíra André, Farah Al-Atrash, Hanan Al-Khatri, Rea Risky Alprianti, Hayder Alsaad, Rucha Amin, Eleni Ampatzi, Alpha Yacob Arsano, Montazami Azadeh, Elie Azar, Bannazadeh Bahareh, Amina Batagarawa, Susanne Becker, Carolina Buonocore, Bin Cao, Joon-Ho Choi, Chungyoon Chun, Hein Daanen, Siti Aisyah Damiati, Lyrian Daniel, Renata De Vecchi, Shivraj Dhaka, Samuel Domínguez-Amarillo, Edyta Dudkiewicz, Lakshmi Prabha Edappilly, Jesica Fernández-Agüera, Mireille Folkerts, Arjan Frijns, Gabriel Gaona, Vishal Garg, Stephanie Gauthier, Shahla Ghaffari Jabbari, Djamila Harimi, Runa T. Hellwig, Gesche M. Huebner, Quan Jin, Mina Jowkar, Renate Kania, Jungsoo Kim, Nelson King, Boris Kingma, M. Donny Koerniawan, Jakub Kolarik, Shailendra Kumar, Alison Kwok, Roberto Lamberts, Marta Laska, M. C. Jeffrey Lee, Yoonhee Lee, Vanessa Lindermayr, Mohammadbagher Mahaki, Udochukwu Marcel-Okafor, Laura Marín-Restrepo, Anna Marquardsen, Francesco Martellotta, Jyotirmay Mathur, Gráinne McGill, Isabel Mino-Rodriguez, Di Mou, Bassam Moujalled, Mia Nakajima, Edward Ng, Marcellinus Okafor, Mark Olweny, Wanlu Ouyang, Ana Ligia Papst de Abreu, Alexis Pérez-Fargallo, Indrika Rajapaksha, Greici Ramos, Saif Rashid, Christoph F. Reinhart, Ma. Isabel Rivera, Mazyar Salmanzadeh, Karin Schakib-Ekbatan, Stefano Schiavon, Salman Shooshtarian, Masanori Shukuya, Veronica Soebarto, Suhendri, Mohammad Tahsildoost, Federico Tartarini, Despoina Teli, Priyam Tewari, Samar Thapa, Maureen Trebilcock, Jörg Trojan, Ruqayyatu B. Tukur, Conrad Voelker, Yeung Yam, Liu Yang, Gabriela Zapata-Lancaster, Yongchao Zhai, Yingxin Zhu, Zahra Sadat Zomorodian

**Affiliations:** 1 Building Science Group, Karlsruhe Institute of Technology, Englerstr. 7, 76131 Karlsruhe, Germany; 2 WIN-Kolleg Thermal comfort and pain, Heidelberg Academy of Sciences and Humanities, Karlstr. 4, 69117 Heidelberg, Germany; 3 Mechanical Engineering Department, University of Technology, Iraq, Al sinaea, 10066 Baghdad, Iraq; 4 CTC/Department of Civil Engineering, Federal University of Santa Catarina, Rua João Pio Duarte Silva, s/n. Córrego Grande, 88.040-901 Florianópolis, SC Brazil; 5 School of Architecture and Built Environment, German Jordanian University, Darat Othman Bdeir, 11180 Amman, Jordan; 6 Civil and Architectural Engineering Department, Sultan Qaboos University, Al-Khod, P.O. Box: 33, PC: 123 Muscat, Oman; 7 Laboratory of Building Technology, Department of Architecture, Institut Teknologi Bandung, Ganesha 10, 40132 Bandung, Indonesia; 8 Department of Building Physics, Bauhaus-University Weimar, Coudraystr. 11A, 99423 Weimar, Germany; 9 Energy & Climate Change Group, Faculty of Engineering & Physical Sciences, University of Southampton, University Road, SO17 1BJ Southampton, United Kingdom; 10 Welsh School of Architecture, Cardiff University, King Edward VII Avenue, CF10 3NB Cardiff, United Kingdom; 11 Department of Architecture, Massachusetts Institute of Technology, Massachusetts Ave, 02139 Cambridge, United States of America; 12 Faculty of Engineering, Environment and Computing, BNE Research Centre, Coventry University, Gulson road, CV1 2JH Coventry, United Kingdom; 13 Industrial and Systems Engineering, Khalifa University, Masdar Campus, Building 1B, 54224 Abu Dhabi, United Arab Emirates; 14 Architecture, University of Tehran, Kish International Campus, Niyayesh St., Mirmohanna Blvd, 79416-55665 Kish, Iran; 15 Department of Architecture, Ahmadu Bello University, Zaria, Nigeria, Samaru, Zaria Nigeria; 16 Department of Cognitive and Clinical Neuroscience, Central Institute of Mental Health, Medical Faculty Mannheim, Heidelberg University, J5, 68159 Mannheim, Germany; 17 CTC/Department of Architecture and Urban Planning, Federal University of Santa Catarina, Campus Trindade, 88040-970 Florianópolis, SC Brazil; 18 Department of Building Science, School of Architecture, Tsinghua University, Shuangqing Road, 100084 Beijing, China; 19 School of Architecture, University of Southern California, 850 Bloom Walk, Watt Hall 204, 90089-0291 Los Angeles, California United States of America; 20 Department of Interior Architecture and Built Environment, Yonsei University, 50 Yonsei-ro, 03722 Seoul, Republic of Korea; 21 Department of Human Movement Sciences, Faculty of Behavioral and Movement Sciences, Amsterdam Movement Sciences, Vrije Universiteit Amsterdam, Van der Boechorststraat 7, 1081 BT Amsterdam, The Netherlands; 22 School of Architecture and Built Environment, The University of Adelaide, North Terrace, 5005 Adelaide, Australia; 23 Indian Green Building Council (IGBC), Confederation of Indian Industry (CII), Survey#64, 500084 Hyderabad, India; 24 Instituto Universitario de Arquitectura y Ciencias de la Construcción, Escuela Técnica Superior de Arquitectura, Universidad de Sevilla, Avenida de Reina Mercedes, 2, 41012 Sevilla, Spain; 25 Faculty of Environmental Engineering, Wroclaw University of Science and Technology, Wybrz. Wyspianskiego 27, 50-370 Wroclaw, Poland; 26 Department of Civil Engineering, Building Technology and Construction Management, Indian Institute of Technology Madras, Building Sciences Block, 600036 Chennai, India; 27 Department of Mechanical Engineering, Eindhoven University of Technology, Groene Loper 3, 5612AE Eindhoven, The Netherlands; 28 Departamento de recursos hídricos y ciencias ambientales, Universidad de Cuenca, Av. 12 de Abril, 10203 Cuenca, Ecuador; 29 Centre for IT in Building Science, International Institute of Information Technology (IIIT), Gachibowli, 500032 Hyderabad, India; 30 Faculty of Architecture and Urbanism, Tabriz Islamic Art University, Azadi, 5164736931 Tabriz, Iran; 31 Faculty of Engineering, Universiti Malaysia Sabah, Jalan UMS, 88400 Kota Kinabalu, Malaysia; 32 Architecture, Design and Media Technology, Aalborg University, Rendsburggade 14, 9000 Aalborg, Denmark; 33 Energy Efficiency Design, Augsburg University of Applied Sciences, An der Hochschule 1, 86161 Augsburg, Germany; 34 Energy Institute, University College London, 14 Upper Woburn Place, WC1H 0NN London, United Kingdom; 35 Department of Architecture and Civil Engineering, Chalmers University of Technology, Sven Hultins gata 6, SE-412 96 Gothenburg, Sweden; 36 Faculty of Engineering, Environment and Computing, Coventry University, Gulson road, CV1 2JH Coventry, United Kingdom; 37 School of Architecture, Design and Planning, The University of Sydney, Wilkinson Bldg G04, 2006 Sydney, Australia; 38 Training and Performance Optimization, The Netherlands Organisation for Applied Sciences, Kampweg 55, 3769 DE Soesterberg, The Netherlands; 39 Department of Nutrition, Exercise and Sports, University of Copenhagen, 2100 Copenhagen, Denmark; 40 Department of Civil Engineering, Technical University of Denmark, Brovej 118, 2800, Kgs, Lyngby, Denmark; 41 Centre for Energy and Environment, Malaviya National Institute of Technology (MNIT), JLN Marg, 302006 Jaipur, India; 42 Department of Architecture, College of Design, University of Oregon, 1206 University of Oregon, 97403 Eugene, United States of America; 43 Department of Interior Design, National Taichung University of Science and Technology, 129, Sec. 3, Sanmin Road, 40401 Taichung, Taiwan; 44 Mechanical Engineering, Shahid Bahonar University of Kerman, Jomhouri, 76169133 Kerman, Iran; 45 Architecture, Federal Polytechnic, Polytechnic Road, 460262 Nekede, Nigeria; 46 Faculty of Architecture, Construction and Design, University of Bio-Bio, Avda Collao 1202, 4081112 Concepcion, Chile; 47 Department of Psychology, University of Koblenz-Landau, Fortstraße 7, 76829 Landau, Germany; 48 Dipartimento di Scienze dell’Ingegneria Civile e dell’Architettura, Politecnico di Bari, Via Orabona, 4, 70125 Bari, Italy; 49 Mackintosh Environmental Architecture Research Unit, Glasgow School of Art, 167 Renfrew Street, G3 6RQ Glasgow, United Kingdom; 50 Institute for Environmental Design and Engineering, University College London, Gower Street, WC1E 6BT London, United Kingdom; 51 Project-team BPE, Cerema, 46 Rue St Théobald, 38081 L’Isle d’Abeau, France; 52 Center for the Built Environment (CBE), University of California, Berkeley, 232 Wurster Hall #1800, 94720 Berkeley, United States of America; 53 School of Architecture, Institute of Future Cities, Institute of Environment, Energy and Sustainability, The Chinese University of Hong Kong, Shatin, N.T., Hong Kong SAR, China; 54 Architecture, Imo State University, Samek Road, 460222 Owerri, Nigeria; 55 Faculty of the Built Environment, Uganda Martyrs University, Nkozi, Uganda; 56 School of Architecture, The Chinese University of Hong Kong, Shatin, N.T., Hong Kong SAR, China; 57 Department of Civil Construction, Federal Institute of Santa Catarina, Av. Mauro Ramos, 950 - Centro, 88.020-300 Florianópolis, SC Brazil; 58 Department of Building Science, University of Bio-Bio, Avda Collao 1202, 4081112 Concepcion, Chile; 59 Department of Architecture, University of Moratuwa, 10400 Moratuwa, Sri Lanka; 60 Department of Building Physics/Low Energy Buildings, Technical University Kaiserslautern, Paul-Ehrlich-Straße 29, 67663 Kaiserslautern, Germany; 61 Departamento de Arquitectura, Facultad de Arquitectura, Urbanismo y Geografía, Universidad de Concepción, Barrio Universitario, Casilla 160-C, 4089100 Concepción, Chile; 62 Property, Construction and Project Management, RMIT University, Swanston, 3000 Melbourne, Australia; 63 Department of Restoration Ecology and Built Environment, Tokyo City University, Ushikubo-Nishi 3-3-1, 224-8551 Yokohama, Japan; 64 Department of construction, Shahid Beheshti University, Evin, 1983969411 Tehran, Iran; 65 Sustainable Buildings Research Centre, University of Wollongong, Squires Way, 237, 2500 Wollongong, Australia; 66 Division of Building Services Engineering, Department of Architecture and Civil Engineering, Chalmers University of Technology, Sven Hultins gata 6, SE-412 96 Göteborg, Sweden; 67 Department of Environmental Studies, Salesian College, 734219 Sonada, Darjeeling India; 68 Department of Architectural Theory and Design, University of Bio-Bio, Avda Collao 1202, 4081112 Concepción, Chile; 69 Akademie für angewandte Bewegungswissenschaften gGmbH, Walter-Krause-Str. 11, 68163 Mannheim, Germany; 70 Department of Mechanical and Automation Engineering, The Chinese University of Hong Kong, Shatin, N.T., Hong Kong SAR, China; 71 School of Architecture, Xi’an University of Architecture and Technology, Yanta Road, 710055 Xi’an, China; 72 Welsh School of Architecture, Cardiff University, King Edward VII Av., CF10 3NB Cardiff, United Kingdom

**Keywords:** Psychology and behaviour, Civil engineering

## Abstract

Thermal discomfort is one of the main triggers for occupants’ interactions with components of the built environment such as adjustments of thermostats and/or opening windows and strongly related to the energy use in buildings. Understanding causes for thermal (dis-)comfort is crucial for design and operation of any type of building. The assessment of human thermal perception through rating scales, for example in post-occupancy studies, has been applied for several decades; however, long-existing assumptions related to these rating scales had been questioned by several researchers. The aim of this study was to gain deeper knowledge on contextual influences on the interpretation of thermal perception scales and their verbal anchors by survey participants. A questionnaire was designed and consequently applied in 21 language versions. These surveys were conducted in 57 cities in 30 countries resulting in a dataset containing responses from 8225 participants. The database offers potential for further analysis in the areas of building design and operation, psycho-physical relationships between human perception and the built environment, and linguistic analyses.

## Abstract (summary)

**Table:** 

Measurement(s)	Natural Language • Demographics • humidity • response to temperature stimulus • temperature of air • geographic location • room temperature ambient air
Technology Type(s)	Survey • sensor
Sample Characteristic - Organism	Homo sapiens
Sample Characteristic - Environment	building
Sample Characteristic - Location	Oman • Kingdom of Spain • Ecuador • Chile • Malaysia • Iraq • Poland • Nigeria • Iran • Italy • United States of America • Germany • Brazil • Jordan • Kingdom of the Netherlands • Greece • Sweden • Kingdom of Denmark • India • Sri Lanka • Uganda • South Korea • Republic of China • United Arab Emirates • Australia • French Republic • United Kingdom • Japan • Indonesia • China

Machine-accessible metadata file describing the reported data: 10.6084/m9.figshare.9805289

## Background & Summary

Occupants have a significant influence on their indoor environment and its energy use through their presence and interactions with the building envelope and control system^1–5^. Factors driving occupants-building interactions are linked to either the intention to adjust indoor environmental parameters (e.g. relating to thermal (dis-)comfort), or to non-environmental factors such as leaving the room^6^. The perception of the thermal indoor environment is one important driving factor for actions, including adjustment of heating or cooling set points, or opening windows^6^, which can be described by the adaptive principle: “If a change occurs that produces discomfort, people tend to act to restore their comfort”^7^. Hence, understanding thermal (dis-)comfort is crucial for appropriate design decisions and choosing suitable operation modes in buildings.

According to the widely-used definition by the American Society for Heating, Refrigerating and Air-Conditioning Engineers (ASHRAE), “Thermal comfort is the condition of mind that expresses satisfaction with the thermal environment and is assessed by subjective evaluation”^8^. Consequently, rating scales are often used for this assessment. Whether a specific set of thermal conditions can be considered comfortable is commonly determined via simple thermal sensation ratings (“cold” to “hot”)^9^. In parallel, additional dimensions of thermal perception are known and applied^10^; e.g. affective evaluation (“comfortable” to “extremely uncomfortable”), thermal preference (“cooler” to “warmer”), or personal acceptance (“generally acceptable” to “generally unacceptable”).

**Fig. 1:**
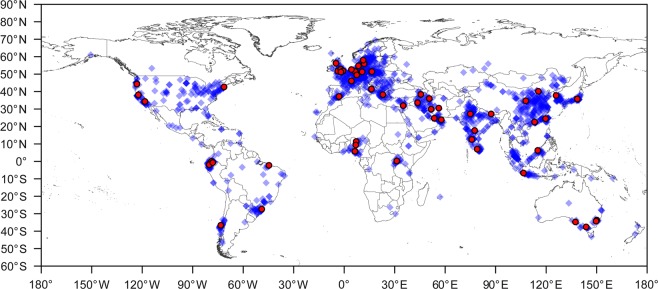
Applications and participants. Places of application of questionnaires (red dots) and places of origin of the 8225 participants (blue diamonds) in this study.

The Scales Project aims at investigating participants’ concept relating to verbal anchors of thermal sensation, thermal comfort, and thermal acceptance scales and to review the validity of existing assumptions (see below) regarding the interpretation of responses on these scales. The dataset consists of data from a large-scale international survey applying a newly developed questionnaire, which asks survey participants to state their perceived distance between the verbal anchors. The questionnaire was applied in 21 language versions. Surveys were conducted in 57 cities in 30 countries resulting in a dataset encompassing responses from 8225 participants (Fig. 1). Because individual inputs are available for each dimension of thermal perception, potential analyses and their statistical power benefit from the within-subject nature of this questionnaire.

Following the project’s objective, this dataset can be used to analyse the conceptual relationships between verbal anchors of one scale, or between one or more scales. For example, thermal sensation is most frequently assessed using the seven-point ASHRAE thermal sensation scale, with the verbal anchors “cold”, “cool”, “slightly cool”, “neutral”, “slightly warm”, “warm”, and “hot”. A common assumption related to the application of the thermal sensation scale is the assumption of equidistance, meaning the difference between “warm” and “hot” is equal to that between “warm” and “slightly warm”. However, research has questioned this assumption^9,11–14^. Hence, the applicability of statistical methods relying on them (e.g. linear regression) needs to be questioned. Beyond reviewing the validity of this assumption, the newly developed questionnaire also enables to analyze the influence of different contexts (e.g. language, climate, and season) and characteristics of individuals (e.g. sex). Further assumptions existing for other dimensions of thermal perception, here: thermal comfort and thermal acceptance, can be assessed also.

Another important assumption in the field of thermal comfort to be reviewed through our dataset, postulates that occupants would be satisfied with the indoor thermal conditions, if they chose one of the middle three verbal anchors of the ASHRAE thermal sensation scale (“slightly cool”, “neutral”, or “slightly warm”). In other words, ‘neutrality’ is assumed to be a desired condition. Various studies have shown individual and contextual differences not supporting this assumption^9,12,13,15–17^. In particular, researchers repeatedly identify a discrepancy in users who declare satisfaction while feeling warm or cool^18–21^.

In addition, the data can also be used for traditional thermal comfort explorations, with more than 5,031 of the datasets include at least one measurement of indoor temperature. Furthermore, the detailed description and availability of questionnaires, available in multiple languages, can serve as a benchmark for future thermal comfort studies and permit replication to other contexts, for example libraries or offices, or other cohorts such as office workers or older people.

## Methods

### Questionnaire development and pilot study

Within the framework of IEA EBC Annex 69, an international and interdisciplinary group consisting of 7 independent research groups in 6 countries (Australia, China, Germany, Korea, Sweden, and United Kingdom) – the initial core group – developed the methods applied in the present paper, based on promising results from an experimental study^14^. This work included several rounds of face-to-face discussions email conversations as well as an online survey. The details of these discussions together with the description of the methods were submitted and registered to the Open Science Framework (OSF) as a pre-analysis plan (PAP)^22^. At the time of submission of the PAP, one application of the questionnaire had been conducted, but the corresponding questionnaires were securely stored and untouched until the moment of submission of the PAP.

The initial core group also developed the questionnaire in an English version. Mandarin (with simplified Chinese), German, Korean and Swedish translations were subsequently prepared by native language experts familiar with the concepts used in the questionnaire.

Each group piloted the initial version of the questionnaire according to the following procedure: The questionnaire was applied without further explanation to at least 7 individuals, of which 2 had to be experienced in the field of human thermal comfort. After collecting the questionnaires, researchers discussed with the participants the length of the questionnaire, the clarity of instructions, and issues when filling out the questionnaire. The observations made through these applications were discussed among the core group and reflected in the revised and final versions of the questionnaire.

### Expansion of research group

After agreeing on a final version of the questionnaire and submitting the PAP, the initial core group reached out to other researchers in the field through existing networks, such as the Network for Comfort and Energy Use in Buildings (NCEUB) (http://nceub.org.uk/), and personal contacts. Additional researchers had to sign a co-author agreement and guarantee to follow the procedures prescribed for data collection (see below). In case researchers used other languages than the above mentioned, they had to translate the questionnaire into their language following the same procedure as initially applied – including piloting the new language version with at least 7 individuals beforehand. The number of 7 individuals was based on observations by the initial core group during the first pilot phase revealing that the number of issues raised by test participants concerning the questionnaire do not increase substantially with a higher number of initial respondents. In addition, the project leader checked the questionnaire with respect to the formalities. The final consortium consisted of 56 research groups from 30 countries with a total of 94 individual researchers.

### Survey participants

Respondents were university students attending lectures, because they were expected to have only minor variations in age and activity level, supporting the focus on our targeted contextual differences. It was a requirement that the students had not participated in lectures addressing the concept of human thermal comfort. Each respondent could only participate once.

### Questionnaire

**Fig. 2:**
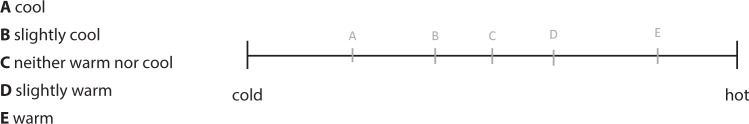
Exemplary response to one of the free-positioning tasks used in the main part of the questionnaire. Participants were asked to position the verbal anchors shown to the left on the empty line with verbal anchors at extreme positions only. Grey lines and letters present the drawing by one participant. The complete question together with instructions and examples for participants is available online^23^.

The questionnaire consists of an introductory page, the two-page main part dealing with the scales and a fourth page addressing the respondents’ background and current thermal state (see all language versions in the online repository sites^23^). The main part used a newly developed free-positioning task, where participants were asked to position the verbal anchors on a straight line (Fig. 2). In the questionnaire scales relating to thermal sensation, thermal comfort and thermal acceptance were investigated. The first questions prompted participants to process each of these scales individually. Later questions addressed the relationship between (1) thermal sensation and thermal comfort; and (2) thermal sensation and thermal acceptance. Verbal anchors were chosen according to ISO 10551^10^. ISO 10551 and many thermal comfort studies also use a preference scale ranging for example from “prefer cooler” to “prefer warmer”. This scale was not used for this study because pilot studies suggested that this scale tends to be misinterpreted by respondents as also pointed out earlier^24^.

In addition to questions relating to thermal sensation, thermal comfort and thermal acceptance scales (Part 1 of the questionnaire), respondents were asked about their current thermal state and background (Part 2 of the questionnaire). Countries and cities of participants’ origin and residence were collated to identify potential adaptations to climatic conditions at the locations where the questionnaires were administered and where participants were living beforehand. See Online-only Table 1 for a full list of variables included in dataset and their source.

### Survey procedure

In each country the questionnaire had to be distributed at least twice during two distinct seasons. The requirement for two distinct seasons was lowered for places with only minor variations in outdoor weather conditions throughout the year. Data were collected from a minimum of 100 respondents per country (a minimum of 50 per season).

The following conditions had to be followed for the distribution of the questionnaires:

- Timing: at the end or if necessary during classes, when participants had been seated at least 30 minutes
- Form: paper-pencil
- Language: local language (in case of large groups of foreigners in a country/class (e.g. Chinese in Korea), researchers were free to distribute more than one language version.

On a separate sheet, researchers noted the following additional information:

- City and Country of survey
- Date, start time of distribution and end time of collection
- Number of questionnaires distributed
- Number of questionnaires received back
- Observations made during survey distribution and collection: e.g. “very high noise levels” or “at day of survey it was unnaturally warm for this time of the year”
- Classification of season: This classification was done without any predefined categories and based on individual researchers’ decision. The researchers used terms for seasons according to typical terms used at their location. Future users of this dataset, who may plan to include such variable into their analysis, can decide whether they follow the classifications given in the dataset or create their own classifications e.g. based on prevailing outdoor conditions, the date of application, KG class, or other information.

In addition, researchers acquired data of the outdoor conditions (outdoor temperature and humidity) from close-by weather stations (either owned by the researchers, available to researchers, or using public sources) and optionally recorded the indoor conditions during the distribution period. Despite their significant influence on thermal perception, indoor conditions were made optional for the following two reasons.

For the first, the main purpose of the Scales Project was not that of a classic thermal comfort study aiming at the analysis of the relationship between indoor thermal conditions (and other factors) and thermal perception assessed through thermal perception scales. The main objective of this study was to reveal participants’ understanding and interpretation of verbal anchors on the scales. The assumption was that they were affected by the prevailing conditions such as seasonal differences or immediate outdoor conditions as well as by an individual’s actual thermal state.

For the second, the methodological intention was to maintain a low level of constraints for additional researchers to join this project. Given the aim of a large response rate from a variety of climates and geographical contexts, a decision was made that the availability of measurement equipment should not be a prerequisite for joining. In addition, classical thermal comfort analysis requires the measurement of indoor air temperature, radiant temperature, relative humidity, and air velocity together with the assessment of clothing insulation level. Due to the place of application being university class rooms, temperature distributions in terms of air temperature and also mean radiant temperature could be expected to vary largely among individual positions in a large classroom, e.g. close-by or further away from windows or air outlets. Measuring thermal conditions at each participants’ seat would have required substantial amount of equipment significantly limiting the number of participating researchers.

Therefore, a decision was made to have indoor thermal conditions not mandatory and to focus on the assessment of participants’ self-reported thermal state. Future users of this data set should be aware of the limitations. Those planning to use recorded indoor air temperatures can still use large parts of the dataset, as 5,031 questionnaires include at least one measurement of indoor air temperature.

### Data preparation

Individual research groups prepared the data from their questionnaires and submitted for each application two files: one containing the data transferred from the questionnaire, one containing the additional information for each application. The positions of the labels drawn in the free positioning task were quantified using a ruler and measuring the distance of the positioned label to the left end of each horizontal line.

Upon reception of a dataset, the project leader validated the dataset by means of an automated script (see section Technical validation). In addition, the project leader made the following adjustments to harmonize the data and added further variables by means of an R script available online^23^ (see section “Custom code used” below):

Adjustments:

- Researchers participating in this study were advised to print the questionnaire, so that each line representing a linear scale was exactly 100 mm long. This would result in measured distances of verbal anchors between 0 mm and 100 mm. However, there were several cases where the printouts were slightly distorted, i.e. shorter or longer. The real length of the lines in the printouts was reported with the information for each application. Based on this information, the measured values were adjusted for the ratio between the real length of the line in the printed version and the prescribed length of 100 mm.
- Date and time formats were harmonized.
- Season descriptions were harmonized.
  Additional variables:
- KG class: Koeppen-Geiger (KG) classifications were derived for the place of survey (provided by the researcher), and the places of current residence, previous residence, and origin (as stated by the participants). To obtain the KG class for each combination of city and country, the KG world map (Version March 2017) provided for R (http://koeppen-geiger.vu-wien.ac.at/present.htm) was used. This map is based on data from 1986 to 2010 and is the re-analysed KG map with a resolution of 5 arc minutes using the downscaling algorithms^25^. In order to obtain the KG class automatically, the latitude and longitude were first derived based on Nominatim, the search engine for OpenStreetMap data (http://nominatim.openstreetmap.org), then converted to the pixel number of the map.
- Language type: The verbal anchors differ in their type between languages (see also^10^). In some languages, e.g. English, two adjectives are used on the cool and warm side of the scale, respectively, e.g. “warm” and “hot”. Data entries from these languages were assigned the language type “2”. Other languages, e.g. Portuguese, use only one adjective on each side, e.g. “frio”. These are language type “1”. In addition, few languages use either two adjectives on the hot side and one on the cold side (“3h”) or vice versa (“3c”).
- Adaptation level: Depending on the answers to the places of current and previous residence, this variable had the levels: “low”, “middle”, or “high”. The coding was based on the length of residency and the KG classes of current and previous place of living. “Low” denotes that the respondent was living less than a year in the current KG class and that the previous place had a different KG class. “Middle” was assigned to those living 1 to 3 years in the current KG class, but a different one before. All others, i.e. living more than 3 years in the current KG class are “high”.
- Native: The variable native speaker was a binary variable (yes/no) generated. Responses are marked as “yes”, in case the language of the questionnaire is equal to (one of) the language(s) spoken in the country of origin of the respondent. All other responses were marked as “no”.
- Country of residence plausible: Participants reported their country of residence. This record was compared to the country of application noted by the researcher. In case these two countries differed (52 responses), the new variable “Country of residence plausible” was set to “no”, otherwise “yes”.

The variables available in the dataset, including their measurement scale, and levels (if applicable) are presented in Online-only Table 1.

### Ethics and consent

Ethic approvals were acquired where institutional or national requirements made it necessary, such as the Institutional Review Board’s approval. Informed consent was obtained from all subjects before conducting the survey.

## Data Records

All data records listed in this section are available from the project page^23^ on OSF and can be downloaded without an OSF account. The information regarding the cities of current residence, previous residence and origin were removed, as they can serve to identify an individual participant. The R script used for pre-processing the semi-raw data is also available. The data were licensed under a CC0 1.0 Universal license.

### Data structure

#### All Datasets

File format: comma separated values file (.csv).

These files contain:

- Individual raw datasets without information on cities
  - survData: file containing participants responses
  - survInfo: file containing researcher observations
  - summary reports: Summary report by researcher for each application

- One changelog-File: Record of changes made to raw datasets
- Codebooks for each raw data
- Combined dataset including all information from survData and survInfo file together with additional variables.

#### Questionnaires

File format: Adobe pdf (.pdf).

These files contain the 21 language versions used.

#### Data templates

File format: Microsoft Excel (.xlsx).

These files contain the data templates used for data collection.

#### R Scripts

File format: plain text files (.R)

These files contain the R Scripts used for processing the raw datasets, technical validation, and additional variables.

## Technical Validation

Incoming datasets were rigorously checked in several steps:

1. Visual inspection of the datasets, whether they comply with the prescribed formats. If not, datasets were send back to researchers.
2. Semi-automated R Script to validate
  - Spelling of country names, city names, and language codes
  - Whether KG class can be derived automatically (otherwise KG class was added manually)
  - Whether data points are within the expected range (e.g. relative humidity between 0 and 100%), or one of the available categories (e.g. only one of 7 age groups)
3. Additional checks. After combining all datasets, combinations of data points were validated. For example, it can be expected that the verbal anchors of the sensation scale are drawn in the right order, i.e. from cold to hot. Responses, where the verbal anchors for the sensation scale were not in this order were flagged. In addition, outliers were flagged in case a multi-variate regression analysis detected them as outliers. The project leader informed researchers of those data points being flagged and requested an additional check of the original questionnaire. These validations looking for data consistency revealed that the answer patterns in questions 1a, 2a, and 3a, for 266, 307, and 84 questionnaires, respectively, did not meet the researchers’ expectations. The additional checks showed, that one researcher did not understand parts of the instruction of data entry correctly and repeated the data entry again. In the other cases, only 49, 34, 5 values were not correctly transferred, i.e. in 81.6%, 88.9%, 94%, the data was correctly transferred. In case there were more than 10% of data points flagged per one application, the project leader checked the validity of data entries at a random base based on scans of original questionnaires provided by the submitting researchers.

## Usage Notes

For further analyses, it is recommended to use the final dataset on OSF and not the individual raw datasets, because revisions by data providers have only be made on the final dataset.

In total, 9111 questionnaires were distributed and 8225 responses collected (90% response rate). Note that the dataset provided consists of all 8225 questionnaires submitted. The authors did not want to exclude any questionnaire from the dataset based on specific exclusion criteria, e.g. outlier definition or completeness of questionnaire. Future users of the dataset can make their own decisions, which questionnaires they consider as valid or not. Any exclusion criteria from the authors side, would limit the freedom of future users to make such decision.

## Data Availability

R software^26^ was used for all steps requiring data manipulation described above in section Data preparation and below in section Technical validation. Custom code was developed for these steps and is made available (see Code availability below). The custom code consists of three main scripts with additional functions loaded when running the scripts. The first script (ScalesSurv_00_DataPreparationAndChecks.r) contains the steps for initial data screening and calculating additional variables for individual datasets. The second script (ScalesSurv_01_LoadAllDatasets_compl_final.R) combines individual datasets and harmonizes some factor levels, e.g. changing all season descriptors “fall” to “autumn” for consistency with other descriptors. The third script (ScalesSurv_02_Prepare_Data_final.R) is used for additional data preparations, such as additional harmonisations and adjustments to individual data points based on the results from technical validation. All R scripts used for validating incoming data, acquiring climate classifications, and calculating additional variables are available on OSF^23^. The R version used for processing the data was 3.5.2 (2019-04-23). The packages used for data preparation and validation were: knitr, gridExtra, grid, ggplot2, pander, xtable, png, kableExtra, reshape2, SparseM, plyr, data.table.

## Online-only Table

**Online-only Table 1:** Variables their categories, subcategories, measurement scales, and levels. Subcategories are according to the ontology for building monitoring data^27^.

Variable name	Category	Sub-category	Description	Measurement scale	Number of levels	Level descriptor
Adapt	Inhabitants	Attributes	Adaptation level	Ordinal	3	1: living less than 1 year in current climate region; 2: 1 – 3 years; 3: > 3 years
Age	Inhabitants	Attributes	Age group	Ordinal	6	1: <21 years old; 2: 21‐25 years; 3: 26–30 years; 4: 31–35 years; 5: 36–40 years; 6: >40
KG Residence	Inhabitants	Attributes	KG classification of participants place of residence^a^	Nominal	20	Af, Am, As, Aw, BSh, BSk, BWh, BWk, Cfa, Cfb, Cfc, Csa, Csb, Cwa, Cwb, Dfa, Dfb, Dfc, Dwa, ET ^a)^
KG Before Residence	Inhabitants	Attributes	KG classification of place where participants lived before current place of residence^a^	Nominal	22	Af, Am, As, Aw, BSh, BSk, BWh, BWk, Cfa, Cfb, Cfc, Csa, Csb, Cwa, Cwb, Dfa, Dfb, Dfc, Dsb, Dwa, Dwb, ET^a^
KG Origin	Inhabitants	Attributes	KG classification of place where participants grew up^a^	Nominal	24	Af, Am, As, Aw, BSh, BSk, BWh, BWk, Cfa, Cfb, Cfc, Csa, Csb, Cwa, Cwb, Dfa, Dfb, Dfc, Dsb, Dsc, Dwa, Dwb, Dwc, ET^a^
Language	Questionnaire	Attributes	Questionnaire language	Nominal	21	ar, da, de, el, en, esc, ese, ess, fa, fr, id, it, ja, ko, ms, pl, pt, si, sv, tw, zh ^b)^
Language type	Questionnaire	Attributes	group of language with respect to the number of adjectives used for the sensation scale	Nominal	4	1: one adjective for cool and warm side each (e.g. slightly warm, warm, and very warm) 2: two adjectives for cool and warm side each (e.g. slightly warm, warm, and hot) 3c: two adjectives on the cold side and one adjective on warm side of sensation scale 3h: two adjectives on the warm side and one adjective on the cold side of sensation scale
Native	Inhabitants	Attributes	Is participant native speaker of survey language	Binary	2	Yes, no
RHout Survey	External conditions	Hygro-thermal	Outdoor relative humidity at time of survey [°C]	Continuous	—	—
RHout Day	External conditions	Hygro-thermal	Mean relative humidity of day of survey [°C]	Continuous	—	—
RHout Week	External conditions	Hygro-thermal	Weighted mean relative humidity of 7 days before survey [°C] ^b)^	Continuous	—	—
Status HVAC	Control systems/devices	HVAC	Status of HVAC system	Ordinal	8	Air conditioned, Air cooled, Heated, Mechanical ventilation, Mixed ventilation, Natural ventilated, No HVAC, unknown
Tout Survey	External conditions	Hygro-thermal	Outdoor temperature at time of survey [°C]	Continuous	—	—
Tout Day	External conditions	Hygro-thermal	Mean outdoor temperature of day of survey [°C]	Continuous	—	—
Tout Week	External conditions	Hygro-thermal	Weighted mean outdoor temperature of 7 days before survey [°C] ^c)^	Continuous	—	—
Season	External conditions	General	Season descriptor as classified by researcher	Nominal	9	autumn, dry, hot, inter-monsoon, monsoon, spring, summer, wet, winter
Sex	Inhabitants	Attributes	Participants’ sex	Nominal	3	Female, Male, other
Q1_Pos1 to Q5_Pos8	Inhabitants	Attributes	Participants’ perceived position of label on scale	Continuous	-	Values from 0 (left) to 100 (right)
TSV	Inhabitants	Attitudes	Observed thermal sensation votes	Ordinal	7	1: Cold; 2: Cool; 3: Slightly cool; 4: Neutral; 5: Slightly warm; 6: Warm; 7: Hot
TCV	Inhabitants	Attitudes	Observed thermal comfort votes	Ordinal	5	1: Comfortable; 2: Slightly uncomfortable; 3: Uncomfortable; 4: Very uncomfortable; 5: Extremely uncomfortable
TAV	Inhabitants	Attitudes	Observed thermal acceptance votes	Ordinal	4	1: Clearly acceptable; 2: Just acceptable; 3: Just unacceptable; 4: Clearly unacceptable
TPV	Inhabitants	Attitudes	Observed thermal preference votes	Ordinal	7	1: Much cooler; 2: Cooler; 3: Slightly cooler; 4: Without change; 5: Slightly warmer; 6: Warmer; 7: Much warmer

^a^for explanations of acronyms see^26^

^b^all acronyms are language codes according to ISO 639‐1^28^ except esc (Chilean Spanish), ese (Ecuadorian Spanish), ess (Spanish Spanish) – also note: pt (Brazilian Portuguese). See also https://en.wikipedia.org/wiki/List_of_ISO_639-1_codes for a complete list.

^c^Calculated according to EN 15251^29^.
