# Publisher Correction: The Scales Project, a cross-national dataset on the interpretation of thermal perception scales

**DOI:** 10.1038/s41597-019-0348-3

**Published:** 2020-01-06

**Authors:** Marcel Schweiker, Amar Abdul-Zahra, Maíra André, Farah Al-Atrash, Hanan Al-Khatri, Rea Risky Alprianti, Hayder Alsaad, Rucha Amin, Eleni Ampatzi, Alpha Yacob Arsano, Montazami Azadeh, Elie Azar, Bannazadeh Bahareh, Amina Batagarawa, Susanne Becker, Carolina Buonocore, Bin Cao, Joon-Ho Choi, Chungyoon Chun, Hein Daanen, Siti Aisyah Damiati, Lyrian Daniel, Renata De Vecchi, Shivraj Dhaka, Samuel Domínguez-Amarillo, Edyta Dudkiewicz, Lakshmi Prabha Edappilly, Jesica Fernández-Agüera, Mireille Folkerts, Arjan Frijns, Gabriel Gaona, Vishal Garg, Stephanie Gauthier, Shahla Ghaffari Jabbari, Djamila Harimi, Runa T. Hellwig, Gesche M. Huebner, Quan Jin, Mina Jowkar, Renate Kania, Jungsoo Kim, Nelson King, Boris Kingma, M. Donny Koerniawan, Jakub Kolarik, Shailendra Kumar, Alison Kwok, Roberto Lamberts, Marta Laska, M. C. Jeffrey Lee, Yoonhee Lee, Vanessa Lindermayr, Mohammadbagher Mahaki, Udochukwu Marcel-Okafor, Laura Marín-Restrepo, Anna Marquardsen, Francesco Martellotta, Jyotirmay Mathur, Gráinne McGill, Isabel Mino-Rodriguez, Di Mou, Bassam Moujalled, Mia Nakajima, Edward Ng, Marcellinus Okafor, Mark Olweny, Wanlu Ouyang, Ana Ligia Papst de Abreu, Alexis Pérez-Fargallo, Indrika Rajapaksha, Greici Ramos, Saif Rashid, Christoph F. Reinhart, Ma. Isabel Rivera, Mazyar Salmanzadeh, Karin Schakib-Ekbatan, Stefano Schiavon, Salman Shooshtarian, Masanori Shukuya, Veronica Soebarto, Suhendri, Mohammad Tahsildoost, Federico Tartarini, Despoina Teli, Priyam Tewari, Samar Thapa, Maureen Trebilcock, Jörg Trojan, Ruqayyatu B. Tukur, Conrad Voelker, Yeung Yam, Liu Yang, Gabriela Zapata-Lancaster, Yongchao Zhai, Yingxin Zhu, Zahra Sadat Zomorodian

**Affiliations:** 1 Building Science Group, Karlsruhe Institute of Technology, Englerstr. 7, 76131 Karlsruhe, Germany; 2 WIN-Kolleg Thermal comfort and pain, Heidelberg Academy of Sciences and Humanities, Karlstr. 4, 69117 Heidelberg, Germany; 3 Mechanical Engineering Department, University of Technology, Iraq, Al sinaea, 10066 Baghdad, Iraq; 4 CTC/Department of Civil Engineering, Federal University of Santa Catarina, Rua João Pio Duarte Silva, s/n. Córrego Grande, 88.040-901 Florianópolis, SC Brazil; 5 School of Architecture and Built Environment, German Jordanian University, Darat Othman Bdeir, 11180 Amman, Jordan; 6 Civil and Architectural Engineering Department, Sultan Qaboos University, Al-Khod, P.O. Box: 33, PC: 123 Muscat, Oman; 7 Laboratory of Building Technology, Department of Architecture, Institut Teknologi Bandung, Ganesha 10, 40132 Bandung, Indonesia; 8 Department of Building Physics, Bauhaus-University Weimar, Coudraystr. 11A, 99423 Weimar, Germany; 9 Energy & Climate Change Group, Faculty of Engineering & Physical Sciences, University of Southampton, University Road, SO17 1BJ Southampton, United Kingdom; 10 Welsh School of Architecture, Cardiff University, King Edward VII Avenue, CF10 3NB Cardiff, United Kingdom; 11 Department of Architecture, Massachusetts Institute of Technology, Massachusetts Ave, 02139 Cambridge, United States of America; 12 Faculty of Engineering, Environment and Computing, BNE Research Centre, Coventry University, Gulson road, CV1 2JH Coventry, United Kingdom; 13 Industrial and Systems Engineering, Khalifa University, Masdar Campus, Building 1B, 54224 Abu Dhabi, United Arab Emirates; 14 Architecture, University of Tehran, Kish International Campus, Niyayesh St., Mirmohanna Blvd, 79416-55665 Kish, Iran; 15 Department of Architecture, Ahmadu Bello University, Zaria, Nigeria, Samaru, Zaria Nigeria; 16 Department of Cognitive and Clinical Neuroscience, Central Institute of Mental Health, Medical Faculty Mannheim, Heidelberg University, J5, 68159 Mannheim, Germany; 17 CTC/Department of Architecture and Urban Planning, Federal University of Santa Catarina, Campus Trindade, 88040-970 Florianópolis, SC Brazil; 18 Department of Building Science, School of Architecture, Tsinghua University, Shuangqing Road, 100084 Beijing, China; 19 School of Architecture, University of Southern California, 850 Bloom Walk, Watt Hall 204, 90089-0291 Los Angeles, California United States of America; 20 Department of Interior Architecture and Built Environment, Yonsei University, 50 Yonsei-ro, 03722 Seoul, Republic of Korea; 21 Department of Human Movement Sciences, Faculty of Behavioral and Movement Sciences, Amsterdam Movement Sciences, Vrije Universiteit Amsterdam, Van der Boechorststraat 7, 1081 BT Amsterdam, The Netherlands; 22 School of Architecture and Built Environment, The University of Adelaide, North Terrace, 5005 Adelaide, Australia; 23 Indian Green Building Council (IGBC), Confederation of Indian Industry (CII), Survey#64, 500084 Hyderabad, India; 24 Instituto Universitario de Arquitectura y Ciencias de la Construcción, Escuela Técnica Superior de Arquitectura, Universidad de Sevilla, Avenida de Reina Mercedes, 2, 41012 Sevilla, Spain; 25 Faculty of Environmental Engineering, Wroclaw University of Science and Technology, Wybrz. Wyspianskiego 27, 50-370 Wroclaw, Poland; 26 Department of Civil Engineering, Building Technology and Construction Management, Indian Institute of Technology Madras, Building Sciences Block, 600036 Chennai, India; 27 Department of Mechanical Engineering, Eindhoven University of Technology, Groene Loper 3, 5612AE Eindhoven, The Netherlands; 28 Departamento de recursos hídricos y ciencias ambientales, Universidad de Cuenca, Av. 12 de Abril, 10203 Cuenca, Ecuador; 29 Centre for IT in Building Science, International Institute of Information Technology (IIIT), Gachibowli, 500032 Hyderabad, India; 30 Faculty of Architecture and Urbanism, Tabriz Islamic Art University, Azadi, 5164736931 Tabriz, Iran; 31 Faculty of Engineering, Universiti Malaysia Sabah, Jalan UMS, 88400 Kota Kinabalu, Malaysia; 32 Architecture, Design and Media Technology, Aalborg University, Rendsburggade 14, 9000 Aalborg, Denmark; 33 Energy Efficiency Design, Augsburg University of Applied Sciences, An der Hochschule 1, 86161 Augsburg, Germany; 34 Energy Institute, University College London, 14 Upper Woburn Place, WC1H 0NN London, United Kingdom; 35 Department of Architecture and Civil Engineering, Chalmers University of Technology, Sven Hultins gata 6, SE-412 96 Gothenburg, Sweden; 36 Faculty of Engineering, Environment and Computing, Coventry University, Gulson road, CV1 2JH Coventry, United Kingdom; 37 School of Architecture, Design and Planning, The University of Sydney, Wilkinson Bldg G04, 2006 Sydney, Australia; 38 Training and Performance Optimization, The Netherlands Organisation for Applied Sciences, Kampweg 55, 3769 DE Soesterberg, The Netherlands; 39 Department of Nutrition, Exercise and Sports, University of Copenhagen, 2100 Copenhagen, Denmark; 40 Department of Civil Engineering, Technical University of Denmark, Brovej 118, 2800, Kgs, Lyngby, Denmark; 41 Centre for Energy and Environment, Malaviya National Institute of Technology (MNIT), JLN Marg, 302006 Jaipur, India; 42 Department of Architecture, College of Design, University of Oregon, 1206 University of Oregon, 97403 Eugene, United States of America; 43 Department of Interior Design, National Taichung University of Science and Technology, 129, Sec. 3, Sanmin Road, 40401 Taichung, Taiwan; 44 Mechanical Engineering, Shahid Bahonar University of Kerman, Jomhouri, 76169133 Kerman, Iran; 45 Architecture, Federal Polytechnic, Polytechnic Road, 460262 Nekede, Nigeria; 46 Faculty of Architecture, Construction and Design, University of Bio-Bio, Avda Collao 1202, 4081112 Concepcion, Chile; 47 Department of Psychology, University of Koblenz-Landau, Fortstraße 7, 76829 Landau, Germany; 48 Dipartimento di Scienze dell’Ingegneria Civile e dell’Architettura, Politecnico di Bari, Via Orabona, 4, 70125 Bari, Italy; 49 Mackintosh Environmental Architecture Research Unit, Glasgow School of Art, 167 Renfrew Street, G3 6RQ Glasgow, United Kingdom; 50 Institute for Environmental Design and Engineering, University College London, Gower Street, WC1E 6BT London, United Kingdom; 51 Project-team BPE, Cerema, 46 Rue St Théobald, 38081 L’Isle d’Abeau, France; 52 Center for the Built Environment (CBE), University of California, Berkeley, 232 Wurster Hall #1800, 94720 Berkeley, United States of America; 53 School of Architecture, Institute of Future Cities, Institute of Environment, Energy and Sustainability, The Chinese University of Hong Kong, Shatin, N.T., Hong Kong SAR, China; 54 Architecture, Imo State University, Samek Road, 460222 Owerri, Nigeria; 55 Faculty of the Built Environment, Uganda Martyrs University, Nkozi, Uganda; 56 School of Architecture, The Chinese University of Hong Kong, Shatin, N.T., Hong Kong SAR, China; 57 Department of Civil Construction, Federal Institute of Santa Catarina, Av. Mauro Ramos, 950 - Centro, 88.020-300 Florianópolis, SC Brazil; 58 Department of Building Science, University of Bio-Bio, Avda Collao 1202, 4081112 Concepcion, Chile; 59 Department of Architecture, University of Moratuwa, 10400 Moratuwa, Sri Lanka; 60 Department of Building Physics/Low Energy Buildings, Technical University Kaiserslautern, Paul-Ehrlich-Straße 29, 67663 Kaiserslautern, Germany; 61 Departamento de Arquitectura, Facultad de Arquitectura, Urbanismo y Geografía, Universidad de Concepción, Barrio Universitario, Casilla 160-C, 4089100 Concepción, Chile; 62 Property, Construction and Project Management, RMIT University, Swanston, 3000 Melbourne, Australia; 63 Department of Restoration Ecology and Built Environment, Tokyo City University, Ushikubo-Nishi 3-3-1, 224-8551 Yokohama, Japan; 64 Department of construction, Shahid Beheshti University, Evin, 1983969411 Tehran, Iran; 65 Sustainable Buildings Research Centre, University of Wollongong, Squires Way, 237, 2500 Wollongong, Australia; 66 Division of Building Services Engineering, Department of Architecture and Civil Engineering, Chalmers University of Technology, Sven Hultins gata 6, SE-412 96 Göteborg, Sweden; 67 Department of Environmental Studies, Salesian College, 734219 Sonada, Darjeeling India; 68 Department of Architectural Theory and Design, University of Bio-Bio, Avda Collao 1202, 4081112 Concepcion, Chile; 69 Akademie für angewandte Bewegungswissenschaften gGmbH, Walter-Krause-Str. 11, 68163 Mannheim, Germany; 70 Department of Mechanical and Automation Engineering, The Chinese University of Hong Kong, Shatin, N.T., Hong Kong SAR, China; 71 School of Architecture, Xi’an University of Architecture and Technology, Yanta Road, 710055 Xi’an, China; 72 Welsh School of Architecture, Cardiff University, King Edward VII Av., CF10 3NB Cardiff, United Kingdom

Correction to: *Scientific Data* 10.1038/s41597-019-0272-6, published online 26 November 2019

During the typesetting process, errors were introduced into the affiliations of authors Maureen Trebilcock and Yoonhee Lee. This has been corrected in both the HTML and PDF versions of this Data Descriptor.

